# The equity and effectiveness of China’s 2009 folic acid supplementation programme for long-term child development

**DOI:** 10.1080/16549716.2026.2691381

**Published:** 2026-06-22

**Authors:** Naiyuan Liang, Cheng Huang, Qiang Pan

**Affiliations:** a School of Economics and Management, Harbin Institute of Technology, Shenzhen, People’s Republic of China; b Institute for Global Health and Development, Peking University, Beijing, People’s Republic of China; c China Center for Health Economic Research (CCHER), National School of Development, Peking University, Beijing, People’s Republic of China; d School of Economics, Peking University, Beijing, People’s Republic of China

**Keywords:** Folic acid supplementation, child development, health equity, quasi-experimental design, socioeconomic status

## Abstract

**Background:**

Maternal folic acid supplementation is a cornerstone of public health for preventing neural tube defects (NTDs), yet its long-term impact on child development remains underexplored.

**Objective:**

This study evaluates the long-term cognitive and mental health associations of maternal folic acid supplementation by utilising China’s 2009 Folic Acid Supplementation Programme for rural women as a quasi-experimental framework.

**Methods:**

We analysed 1,953 children aged 10–15 years from the 2020 China Family Panel Studies. Using provincial NTD prevalence in 2009 from the Maternal and Child Health Surveillance system as a proxy for intervention intensity, we employed difference-in-differences models to estimate associations, adjusting for demographic and parental covariates.

**Results:**

Maternal folic acid supplementation was significantly associated with improved outcomes among girls: higher memory scores (β [95% CI], 0.232 [0.069, 0.394]), enhanced numerical reasoning skills (5.234, [2.188, 8.279]), lower Center for Epidemiological Studies Depression Scale score (−0.523 [−0.933, −0.113]), and reduced likelihood of depressive symptoms (−0.024 [−0.048, −0.001]). No comparable associations were observed in the boys. Although cognitive associations were observed across socioeconomic status (SES) groups among girls, the mental health associations were concentrated among girls from higher-SES families. Results were robust to adjustment for covariates.

**Conclusions:**

This study highlights sex-specific, long-term associations of maternal folic acid supplementation but reveals that socioeconomic disparities may constrain mental health gains. Thus, universal nutrition interventions alone may be insufficient to bridge health equity gaps. The findings should be interpreted within the broader nutritional ecology and alongside concurrent macro-social changes that shape child development outcomes.

## Paper Context

- **Main findings:** Maternal folic acid supplementation was associated with sex-specific, long-term cognitive and mental health outcomes among girls aged 10–15, with mental health associations exclusively concentrated among those from families with higher-socioeconomic status.
- **Added knowledge:** Leveraging a national programme as a quasi-experimental framework, this study provided evidence that the associations of folic acid supplementation may extend beyond birth defect prevention while revealing that within a complex nutrition and psychosocial environment, universal biomedical interventions risk inadvertently exacerbating socioeconomic inequalities in adolescent mental health when implemented in isolation.
- **Global health impact for policy and action:** Where applicable, policymakers in low- and middle-income countries should consider moving beyond ‘one-size-fits-all’ biomedical approaches by integrating nutrition programmes with targeted psychosocial support, to ensure that the potential benefits of public health investments are shared equitably across socioeconomic groups and in alignment with the Sustainable Development Goals.

## Background

Bridging developmental gaps through early-life nutrition interventions is not only a health imperative but also a fundamental strategy for achieving the Sustainable Development Goals (SDGs), particularly SDG 3 (Good Health and Well-Being) and SDG 10 (Reduced Inequalities). Maternal folic acid supplementation is a key strategy to address micronutrient deficiencies and improve maternal and child health outcomes [1]. Neural tube defects (NTDs) are preventable with maternal folic acid supplementation [2–4], and since the 1990s, global recommendations have promoted its use for prenatal health [5]. Beyond this established benefit, emerging research suggests associations with reduced prevalence of limb reduction defects [6], lower risk of autism spectrum disorders [7–10], enhanced cognitive ability [11–13], improved mental health [14], and decreased risk of severe language delay [15]. However, research on the long-term associations of maternal folic acid supplementation with cognitive ability and mental health in healthy children remains limited, particularly on its interaction with child sex and family socioeconomic status (SES). Moreover, most studies have been constrained by methodological challenges, such as small-scale randomised controlled trials with short-term follow-up [12] or observational designs susceptible to residual confounding [11].

China’s 2009 Folic Acid Supplementation Programme for the Prevention of Neural Tube Defects targets rural women of reproductive age (WRA). The programme provides a unique opportunity to examine associations between maternal folic acid supplementation and children’s long-term cognitive ability and mental health through a quasi-experimental framework. By narrowing the nutrition gap in underprivileged rural populations, such initiatives potentially disrupt the intergenerational transmission of disadvantage, directly contributing to SDG 10 (Reduced Inequalities). This policy-driven initiative mitigates selection bias from unobserved factors, such as health awareness, which is a common limitation in observational studies, while providing population-level insights into real-world effectiveness.

Historically, due to limited health awareness and affordability, the usage of folic acid tablets in many rural regions was low. By 2007, NTD prevalence reached 7.15 per 10,000 births in rural areas, 2.2 times higher than urban rates (3.20 per 10,000) [16]. In response, the Chinese government launched the Folic Acid Supplementation Programme in 2009, providing free folic acid tablets to rural WRA to narrow the urban-rural disparity in NTD prevalence. Between 2009 and 2011, the central government dedicated 320 million CNY to this programme, substantially increasing folic acid supplementation coverage among rural WRA members and reaching 23.56 million beneficiaries [17]. This initiative not only reduced NTD prevalence but also demonstrated the potential of large-scale, equitable nutrition interventions in resource-constrained settings.

Using this national programme as the basis for a quasi-experimental evaluation, we examine whether the long-term developmental benefits of such a large-scale public health investment are equitably distributed across child sex and family SES. Our findings address a critical question for global health equity: whether universal nutrition interventions alone can bridge developmental gaps, a core challenge for SDG 10 (Reduced Inequalities). These insights offer a reference for folic acid supplementation policy considerations in other low- and middle-income countries (LMICs) where micronutrient deficiencies remain prevalent and the pursuit of health equity is paramount [18], although careful attention to local health systems, cultural contexts, and data infrastructure is required before direct extrapolation.

## Methods

### Study setting

This study utilised individual-level data from the 2020 wave of the China Family Panel Studies (CFPS) to measure children’s long-term cognitive and mental health outcomes. Initiated by the Institute of Social Science Survey at Peking University in 2010 and followed up every two years, the CFPS is a premier, nationally representative longitudinal survey designed to capture the complex dynamics of contemporary Chinese society. It collects comprehensive and objective data encompassing economic activities, educational outcomes, family dynamics, and health and well-being [19]. The 2020 wave comprised a robust sample of 13,017 households and 51,291 individuals. The CFPS’s rigorous design and extensive coverage provide a reliable foundation for evaluating the long-term impacts of public health policies.

To capture the baseline spatial heterogeneity of the policy intervention, we extracted provincial-level NTD prevalence data for 2009 from China’s Maternal and Child Health Surveillance (MCHS) system. Initiated in the 1980s and formally integrated into a unified national network by the Ministry of Health in 1996, the MCHS represents the world’s largest epidemiological surveillance system for maternal and child health. Supported by sustained central government funding, this three-tier (village, township, and county), systematic network covers more than 100 million individuals. Crucially, the NTD prevalence data generated by this system are officially recognised by the State Council and the National Bureau of Statistics, serving as the definitive basis for national health policymaking. We used this authoritative, dynamically monitored, and internal restricted-access administrative dataset to proxy the provincial-level intervention intensity of the 2009 Folic Acid Supplementation Programme.

The programme provided free folic acid tablets at a standardised dose of 0.4 milligrams per day, to be taken during the periconceptional period from three months before conception through the first three months of pregnancy, consistent with World Health Organization recommendations for NTD prevention [1]. The Chinese government established national intake and compliance targets of 90% and 70%, respectively, among rural WRA members by 2011. Published provincial-level studies documented that actual uptake and compliance rates met or exceeded these targets. In Hebei Province, intake rates reached 85–94% and compliance rates reached 76–85% between 2010 and 2012 [20]; in Jiangxi Province, intake rates were 89–92% and compliance rates were approximately 79% between 2011 and 2013 [21]. The programme was implemented through a grassroot delivery mechanism, with village-level health workers responsible for distributing the folic acid tablets and conducting follow-up visits, providing active structural support for adherence.

### Study design

The study employed a quasi-experimental difference-in-differences (DID) design, leveraging China’s 2009 Folic Acid Supplementation Programme, to examine its long-term associations with children’s cognitive ability and mental health. We used data from the 2020 CFPS to evaluate outcomes for children aged 10–15 years, a range determined by the CFPS survey design wherein the cognitive and mental health modules were administered exclusively to this child and adolescent cohort. This yielded a final analytical sample of 1,953 children born between 2005 and 2010.

Notably, as the 2020 data collection coincided with the SARS-CoV-2 pandemic, the CFPS adopted a mixed-mode survey design. Cognitive assessments were restricted to face-to-face interviews (16% of the participants), and mental health measures included telephone interviews (84%). Consequently, the analyses of cognitive ability outcomes used a restricted sample size compared with the analyses of mental health outcomes.

To construct the DID framework, the analytical sample was stratified into two distinct cohorts based on whether the child’s mother was eligible for the free Folic Acid Supplementation Programme: (i) the policy-eligible group (*n* = 277), comprising rural children born in 2010, whose mothers were targeted by the rural-specific programme and who were potentially exposed to the programme in utero; and (ii) the policy-ineligible group (*n* = 1,676), comprising urban children (structurally ineligible for the rural-targeted policy) and rural children born before 2010 (unaffected due to the year they were born). Because the 2009 programme provided free folic acid exclusively to rural WRA, urban WRA were structurally ineligible, creating a quasi-experimental comparison structure for our analysis.

Furthermore, in the absence of systematically recorded, individual-level longitudinal data on folic acid supplementation rates across all provinces during the early stages of the programme, we utilised $\mathrm{NT}D_{09}$ (NTD prevalence per 10,000 births in 2009) as a continuous proxy measure for the heterogeneous intensity of policy implementation (online Supplemental Table S1). This approach was grounded in the needs-based resource allocation logic of the national initiative. The central government allocated more funding to the provinces with the highest baseline disease burden to support folic acid tablet distribution and grassroots monitoring. Consequently, provinces with higher preexisting NTD prevalence likely received more intensive policy attention and more stringent intervention measures. Thus, the baseline NTD prevalence effectively captures the spatial heterogeneity in implementation rigour across China.

### Study variables

Cognitive ability outcomes were assessed using Rasch-standardised scores from the 2020 CFPS data, comprising the following: (i) a memory test evaluating short-term memory ability, where participants recalled 20 words across two sets (10 words per set), and (ii) a number series test measuring numerical reasoning skills through a two-stage adaptive design (three items per set). Mental health outcomes were evaluated using the following: (i) the Center for Epidemiological Studies Depression Scale (CES-D20) score (20 items; item score: 0–3; total range: 0–60) [22] and (ii) a binary variable for depressive symptoms, defined as a CES-D20 score ≥ 17 [23,24].

We adjusted for demographic covariates, including the following: sex (male = 1, female = 0); hukou status (rural = 1, urban = 0), defined as China’s household registration system; parental age at childbirth (five-tier classification: <20 years = 1, 20–24 years = 2, 25–29 years = 3, 30–34 years = 4, and ≥35 years = 5), and parental education (four-tier classification: primary education or below = 1, junior high-school education = 2, high-school education = 3, and college or above = 4).

### Statistical analysis

We employed a continuous DID design to estimate the dose–response relationship between the intensity of the folic acid supplementation policy and child developmental outcomes. The model specification is as follows:

$$\mathrm{Outcom}e_{i,c,p}=\alpha +\beta \left(\mathrm{Intensit}y_{p}\times \mathrm{PolicyEligibl}e_{i}\right)+\gamma X_{i}+\mu_{c}+\mu_{p}+\varepsilon_{i,c,p}$$

where $\mathrm{Outcom}e_{i,c,p}$ refers to measures of cognitive ability or mental health in 2020 for child i from birth cohort c in province p. $\mathrm{Intensit}y_{p}$ refers to provincial-level policy intervention intensity, proxied by $\mathrm{NT}D_{09}$, the provincial NTD prevalence per 10,000 births in 2009. $\mathrm{PolicyEligibl}e_{i}$ is a binary indicator that equals 1 for children eligible for the policy intervention (those with rural hukou and born in 2010), and 0 otherwise. It captures the target population of China’s 2009 Folic Acid Supplementation Programme. The coefficient of interest, β, captures the effect of a unit increase in policy intensity (equivalent to a reduction of 1 per 10,000 births in provincial NTD prevalence) on the outcomes for policy-eligible children relative to the policy-ineligible group. $X_{i}$ includes covariates such as maternal age, paternal age, maternal education, and paternal education. $\mu_{c}$ and $\mu_{p}$ are birth cohort and birth province-fixed effects, respectively. $\varepsilon_{i,c,p}$ is the error term. All data were analysed using the STATA software (Version 17).

## Results

### Descriptive information

Table 1 presents definitions and summary statistics for all the variables used in the sample of Chinese children aged 10–15 years (*n* = 1,953). The cognitive ability and mental health outcomes showed no evidence of sex differences: memory test score (*p* = 0.93), number series test score (*p* = 0.14), CES-D20 score (*p* = 0.18), and depressive symptoms (*p* = 0.08). The distributions of hukou status (*p* = 0.35), birth year (*p* = 0.78), paternal age (*p* = 0.65), maternal education (*p* = 0.45), and paternal education (*p* = 0.76) were comparable between the sexes. The maternal age distributions differed by child sex (*p* = 0.015).

**Table 1.** Characteristics of the sample of Chinese children by sex [mean (SD) or *n* (%)].

Characteristics	Boys sample^a^ (*n* = 1,028)	Girls sample^b^ (*n* = 925)
**Memory test score**	6.0 (1.7)	6.0 (1.7)
**Number series test score**	517.6 (31.4)	512.3 (34.3)
**CES-D20 score**	10.6 (6.5)	11.0 (7.4)
**Depressive symptoms (%)**	146 (14.2)	158 (17.1)
**Hukou status (%)**		
Rural	874 (85.0)	800 (86.5)
Urban	154 (15.0)	125 (13.5)
**Age in years (%)**		
10	168 (16.3)	154 (16.6)
11	197 (19.2)	169 (18.3)
12	169 (16.4)	162 (17.5)
13	175 (17.0)	142 (15.4)
14	153 (14.9)	154 (16.6)
15	166 (16.1)	144 (15.6)
**Maternal age (%)**		
<20	51 (5.0)	37 (4.0)
20–24	369 (35.9)	344 (37.2)
25–29	303 (29.5)	321 (34.7)
30–34	200 (19.5)	157 (17.0)
≥35	105 (10.2)	66 (7.1)
**Paternal age (%)**		
<20	9 (0.9)	6 (0.6)
20–24	245 (23.8)	228 (24.6)
25–29	357 (34.7)	344 (37.2)
30–34	262 (25.5)	220 (23.8)
≥35	155 (15.1)	127 (13.7)
**Maternal education (%)**		
Primary education or below	407 (39.6)	342 (37.0)
Junior high school education	417 (40.6)	393 (42.5)
High school education	125 (12.2)	106 (11.5)
College or above	79 (7.7)	84 (9.1)
**Paternal education (%)**		
Primary education or below	315 (30.6)	284 (30.7)
Junior high school education	440 (42.8)	403 (43.6)
High school education	179 (17.4)	146 (15.8)
College or above	94 (9.1)	92 (9.9)

SD, standard deviation.

^a^Within this sample, the policy-eligible group (rural children born in 2010) comprised 143 children; the policy-ineligible group (urban children and rural children born in 2005–2009) comprised 885 children.

^b^Within this sample, the policy-eligible group (rural children born in 2010) comprised 134 children; the policy-ineligible group (urban children and rural children born in 2005–2009) comprised 791 children.

### Associations between maternal folic acid supplementation and children’s cognitive ability outcomes

Table 2 presents the associations between maternal folic acid supplementation and the cognitive ability outcomes of Chinese children aged 10–15 years. The analyses revealed consistent, positive associations for both cognitive measures. In the full sample (*n* = 326 for memory; *n* = 327 for number series), higher folic acid intervention intensity was associated with higher memory test scores in both the non-adjusted (β [95% CI], 0.187 [0.097, 0.277], *p* < 0.001) and adjusted models (0.187 [0.100, 0.275], *p* < 0.001). Associations were also observed for number series test scores (Model 1: 3.058 [1.001, 5.115], *p* = 0.004; Model 2: 3.113 [1.124, 5.102], *p* = 0.003).

**Table 2.** Association of maternal folic acid supplementation with cognitive ability outcomes in Chinese children aged 10–15 years.

	Memory test score^b,c^					Number series test score^b,d^				
	*n*	Model 1 (95% CI)	*p*-value	Model 2^a^ (95% CI)	*p*-value	*n*	Model 1 (95% CI)	*p*-value	Model 2^a^ (95% CI)	*p*-value
All	326	0.187	<0.001	0.187	<0.001	327	3.058	0.004	3.113	0.003
		(0.097, 0.277)		(0.100, 0.275)			(1.001, 5.115)		(1.124, 5.102)	
Boys	164	0.152	0.171	0.133	0.275	164	0.065	0.948	0.920	0.443
		(−0.068, 0.373)		(−0.109, 0.375)			(−1.942, 2.072)		(−1.462, 3.303)	
Girls	160	0.211	0.037	0.232	0.006	161	5.854	<0.001	5.234	0.001
		(0.013, 0.409)		(0.069, 0.394)			(3.071, 8.636)		(2.188, 8.279)	

CI, confidence interval.

^a^Adjusted for maternal age, paternal age, maternal education, and paternal education.

^b^The analytical sample for cognitive outcomes was restricted to children who completed face-to-face interviews.

^c^Within this sample, the policy-eligible group (rural children born in 2010) comprised 52 children; the policy-ineligible group (urban children and rural children born in 2005–2009) comprised 274 children.

^d^Within this sample, the policy-eligible group (rural children born in 2010) comprised 52 children; the policy-ineligible group (urban children and rural children born in 2005–2009) comprised 275 children.

Sex-stratified analyses demonstrated differential patterns. Among girls, higher folic acid intervention intensity was associated with higher memory test scores (0.232 [0.069, 0.394], *p* = 0.006) and number series test scores (5.234 [2.188, 8.279], *p* = 0.001) in the adjusted models. Among boys, the analyses showed no clear associations with cognitive ability outcomes, as evidenced by confidence intervals spanning the null values (memory: 0.133 [−0.109, 0.375], *p* = 0.275; number series: 0.920 [−1.462, 3.303], *p* = 0.443).

### Associations between maternal folic acid supplementation and children’s mental health outcomes

Table 3 presents the associations between maternal folic acid supplementation and mental health outcomes among Chinese children aged 10–15 years, indicating consistent inverse associations across measurement approaches. For the full sample (*n* = 1,953), higher folic acid intervention intensity was associated with lower CES-D20 scores in both the non-adjusted (−0.426 [−0.633, −0.220], *p* < 0.001) and adjusted models (−0.411 [−0.626, −0.195], *p* < 0.001). Depressive symptoms showed consistent inverse associations (Model 1: −0.020 [−0.034, −0.005], *p* = 0.010; Model 2: −0.019 [−0.034, −0.004], *p* = 0.015).

**Table 3.** Association of maternal folic acid supplementation with mental health outcomes in Chinese children aged 10–15 years.

	CES-D20 score^b^					Depressive symptoms^b^				
	*n*	Model 1 (95% CI)	*p*-value	Model 2^a^ (95% CI)	*p*-value	*n*	Model 1 (95% CI)	*p*-value	Model 2^a^ (95% CI)	*p*-value
All	1,953	−0.426	<0.001	−0.411	<0.001	1,953	−0.020	0.010	−0.019	0.015
		(−0.633, −0.220)		(−0.626, −0.195)			(−0.034, −0.005)		(−0.034, −0.004)	
Boys	1,028	−0.357	0.038	−0.336	0.059	1,028	−0.014	0.204	−0.012	0.267
		(−0.693, −0.020)		(−0.685, 0.013)			(−0.035, 0.008)		(−0.034, 0.010)	
Girls	925	−0.549	0.014	−0.523	0.013	925	−0.026	0.037	−0.024	0.039
		(−0.985, −0.114)		(−0.933, −0.113)			(−0.050, −0.002)		(−0.048, −0.001)	

CI, confidence interval.

^a^Adjusted for maternal age, paternal age, maternal education, and paternal education.

^b^Within this sample, the policy-eligible group (rural children born in 2010) comprised 277 children; the policy-ineligible group (urban children and rural children born in 2005–2009) comprised 1,676 children.

Notably, the magnitudes of the associations varied by sex, aligning with patterns observed for the cognitive ability outcomes. For girls (*n* = 925), higher folic acid intervention intensity was associated with a lower CES-D20 score (−0.523 [−0.933, −0.113], *p* = 0.013) and reduced likelihood of depressive symptoms (−0.024 [−0.048, −0.001], *p* = 0.039) in the adjusted models. Boys (*n* = 1,028) exhibited no clear association with the CES-D20 score (−0.336 [−0.685, 0.013], *p* = 0.059) or depressive symptoms (−0.012 [−0.034, 0.010], *p* = 0.267) in the adjusted models.

### Socioeconomic inequalities in child developmental outcomes

Given that associations between China’s 2009 Folic Acid Supplementation Programme and cognitive ability or mental health outcomes were observed primarily among rural girls, we focused subsequent analyses on this subgroup. In this section, we examine how these associations varied by socioeconomic factors, including parental education level and per capita household income level.

#### Parental education level

In education-stratified analyses of rural Chinese girls aged 10–15 years, differential patterns emerged across parental education levels (Table 4). Among girls whose mothers had primary education or below, higher folic acid intervention intensity was associated with an improved memory test score (0.187 [0.074, 0.300], *p* = 0.002) and a higher number series test score (6.760 [1.071, 12.450], *p* = 0.021). However, no clear associations with mental health outcomes were observed (CES-D20 score: −0.060 [−0.568, 0.449], *p* = 0.817; depressive symptoms: −0.005 [−0.033, 0.024], *p* = 0.757). In contrast, among girls, with mothers attaining junior high-school education or above, the estimates indicated cognitive gains and mental health benefits, including a lower CES-D20 score (−0.943 [−1.309, −0.577], *p* < 0.001) and reduced probability of depressive symptoms (−0.044 [−0.068, −0.020], *p* = 0.001). Paternal education-stratified analyses yielded comparable patterns, with all models adjusted for parental age and counterpart parental education.

**Table 4.** Association of maternal folic acid supplementation with cognitive ability and mental health outcomes in rural Chinese girls aged 10–15 years, stratified by parental education level.

	Memory test score^b,c^			Number series test score^b,d^			CES-D20 score^e^			Depressive symptoms^e^		
	*n*	Model 3^a^ (95% CI)	*p*-value	*n*	Model 3^a^ (95% CI)	*p*-value	*n*	Model 3^a^ (95% CI)	*p*-value	*n*	Model 3^a^ (95% CI)	*p*-value
**Maternal education**												
Primary education and below	86	0.187	0.002	87	6.760	0.021	333	−0.060	0.817	333	−0.005	0.757
		(0.074, 0.300)			(1.071, 12.450)			(−0.568, 0.449)			(−0.033, 0.024)	
Junior high school education and above	59	0.574	<0.001	59	8.102	0.048	465	−0.943	<0.001	465	−0.044	0.001
		(0.328, 0.820)			(0.085, 16.120)			(−1.309, −0.577)			(−0.068, −0.020)	
**Paternal education**												
Primary education and below	88	0.146	0.026	89	6.956	0.003	275	−0.192	0.519	275	−0.018	0.284
		(0.019, 0.273)			(2.495, 11.418)			(−0.780, 0.397)			(−0.051, 0.015)	
Junior high school education and above	56	0.650	<0.001	56	5.531	0.076	524	−0.776	<0.001	524	−0.032	0.017
		(0.385, 0.915)			(−0.611, 11.674)			(−1.196, −0.357)			(−0.058, −0.006)	

CI, confidence interval.

^a^For maternal education, adjusted for maternal age, paternal age, and paternal education. For paternal education, adjusted for maternal age, paternal age, and maternal education.

^b^The analytical sample for cognitive outcomes is restricted to children who completed face-to-face interviews.

^c^Within this sample, the policy-eligible group (rural children born in 2010) comprised 25 children; the policy-ineligible group (rural children born in 2005–2009) comprised 120 children.

^d^Within this sample, the policy-eligible group (rural children born in 2010) comprised 25 children; the policy-ineligible group (rural children born in 2005–2009) comprised 121 children.

^e^Within this sample, the policy-eligible group (rural children born in 2010) comprised 134 children; the policy-ineligible group (rural children born in 2005–2009) comprised 665 children.

#### Per capita household income level

The 2020 CFPS documented a median per capita household income of ¥11,997.5 (US$1,739 at 2020 exchange rates) among rural women. Table 5 presents per capita household income-stratified analyses of maternal folic acid supplementation intervention intensity and mental health outcomes among rural Chinese girls aged 10–15 years. For girls from households at or below median per capita income (*n* = 399), higher folic acid intervention intensity was associated with a reduced CES-D20 score (−0.284 [−0.561, −0.007], *p* = 0.045), while the estimate for depressive symptoms spanned the null (−0.021 [−0.044, 0.003], *p* = 0.087). In contrast, girls from above-median per capita income households (*n* = 400) showed inverse associations for CES-D20 score (−0.922 [−1.557, −0.286], *p* = 0.005) and decreased probability of depressive symptoms (−0.033 [−0.067, 0.000], *p* = 0.049). These results suggest an income gradient in mental health associations with folic acid policy exposure.

**Table 5.** Association of maternal folic acid supplementation with mental health outcomes in rural Chinese girls aged 10–15 years, stratified by per capita household income level.

	CES-D20 score^b^			Depressive symptoms^b^		
	*n*	Model 2^a^ (95% CI)	*p*-value	*n*	Model 2^a^ (95% CI)	*p*-value
Below or at median	399	−0.284	0.045	399	−0.021	0.087
		(−0.561, −0.007)			(−0.044, 0.003)	
Above median	400	−0.922	0.005	400	−0.033	0.049
		(−1.557, −0.286)			(−0.067, 0.000)	

CI, confidence interval.

^a^Adjusted for maternal age, paternal age, maternal education, and paternal education.

^b^Within this sample, the policy-eligible group (rural children born in 2010) comprised 134 children; the policy-ineligible group (rural children born in 2005–2009) comprised 665 children.

### Sensitivity analyses

We ran a pre-trend test to check for potential differences in the pre-policy trends of cognitive ability and mental health outcomes between policy-eligible and policy-ineligible areas (online Supplemental Table S2 and Figure S1). The results revealed no significant differences in pre-policy trends, supporting the parallel trends assumption that underpins our DID design.

China’s 2009 Folic Acid Supplementation Programme showed differential associations by sexes. To assess the robustness of the estimates, we conducted sensitivity analyses of the girls’ sample. We evaluated variations under two scenarios: (i) redefining the pre-intervention cohort using births during 2006–2009 rather than 2005–2009 and (ii) assuming an alternative policy implementation date of June 2006 (three years prior to the actual rollout). The results of these sensitivity analyses remained unchanged (online Supplemental Table S3), supporting that the observed associations reflected the 2009 policy implementation rather than secular trends or cohort selection artifacts. Further sensitivity analysis using Poisson pseudo-maximum likelihood regression for depressive symptoms yielded risk ratios (online Supplemental Table S4) consistent with the primary linear probability model estimates (Table 3).

## Discussion

### Summary of the main findings

This study leveraged China’s 2009 Folic Acid Supplementation Programme, which targeted rural WRA, as a quasi-experimental framework. The findings provide novel evidence of long-term associations between maternal folic acid supplementation and child development. Our analyses revealed that these associations are shaped by two key factors: child sex and family SES. First, we identified a striking sex-specific pattern. Associations were observed exclusively among girls, who showed improvements in both cognitive ability (short-term memory and numerical reasoning) and mental health, as measured by lower CES-D20 scores and a reduced likelihood of depressive symptoms. No comparable associations were found among the boys. Second, and more importantly, we uncovered a disparity in how these associations were distributed across SES among girls. While cognitive associations were broadly observed across girls regardless of family SES, mental health improvements were concentrated exclusively among girls from higher-SES families, defined by parental educational attainment or per capita household income above the median.

### Interpretation within nutritional and social contexts

The observed sex-specific pattern of associations highlights the complexity of early childhood cognitive and mental health development, which is shaped by dietary, household food security, and environmental factors. Consequently, these findings cannot be viewed merely as an isolated effect of a single public health intervention. Rather, they should be interpreted within the broader nutritional ecology of early life and the profound social transformations in China during the study period [25].

From a biological standpoint, two candidate mechanisms may help explain why girls, but not boys, showed associations with folic acid exposure intensity. Epigenetic analyses of cord blood samples have identified sex-specific DNA methylation changes associated with maternal folic acid supplementation. The significant changes observed in the IGF2 gene among girls but not boys suggest that folate may exert sex-specific epigenetic effects on genes relevant to brain development [26]. Additionally, female placentas have been shown to exhibit greater adaptive capacity and more efficient nutrient transport under suboptimal conditions, potentially facilitating more effective folate delivery to the developing female brain [27]. A definitive biological mechanism for sex-differential neurodevelopmental responses to folic acid exposure in utero remains to be established.

It is equally plausible, and perhaps more so, that the observed cognitive and mental health associations among girls partly reflect improved social status and increased parental investment in female children during this era. The rollout of the 2009 Folic Acid Supplementation Programme coincided with profound demographic and cultural transformations in China. The cumulative effects of the One-Child Policy (1979–2016) and the subsequent ‘Care for Girls’ campaign (launched in 2006), which provided financial incentives and social support for families with daughters, collectively contributed to redressing gender imbalances and gradually reducing son preference, triggering a historic shift in gender attitudes [28–30]. As daughters became increasingly valued, households likely directed greater postnatal investments into their health, education, and psychological well-being. This structural shift in gender equity may have acted synergistically with the prenatal nutrition intervention, amplifying the observable developmental gains for the girls [31]. The null findings among boys should be interpreted within this broader social context.

The observed SES gradient in mental health associations adds a further dimension to this interpretation. The finding that cognitive associations were broadly distributed across SES groups among girls, while mental health associations were restricted to higher-SES families, suggests that these two developmental domains are governed by distinct pathways. Folic acid, as an essential micronutrient for foetal neurodevelopment [32], may exert a relatively uniform biological influence on cognitive capacity. Mental health outcomes, however, appear to be more contingent on the postnatal social environment. Resource Substitution Theory offers a compelling framework for interpreting these socioeconomic disparities [33,34]. This theory posits that economic and educational resources can substitute for one another to mitigate mental health risks. Specifically, higher-SES families possess greater access to health knowledge, stress-buffering resources, and supportive parenting practices that may amplify the long-term developmental returns of early nutrition exposure, enabling them to translate a potential biological foundation into sustained mental well-being. In lower-SES families where these complementary resources are scarce, biological intervention alone may be insufficient to generate lasting mental health gains.

### Comparison with the existing literature

Our findings on the cognitive and mental health associations of maternal folic acid supplementation align with the broader literature emphasising the critical importance of nutrition interventions during the ‘first 1,000 days’ of life [35,36]. Previous studies have documented positive associations between early-life nutrition supplementation and improvements in physical health, cognitive development, and mental health during childhood [37–41], as well as enhanced occupational opportunities and higher incomes in adulthood [42]. Furthermore, our observation of sex-differential associations further echoes documented patterns in other nutrition programmes [13,31,43].

Our study extends this literature by documenting the differences between cognitive and mental health associations across SES strata within the same study population. Our findings that cognitive associations were broadly distributed across SES groups among girls, while mental health associations were restricted to higher-SES families, suggest that these two domains are governed by distinct pathways with different susceptibilities to the postnatal social environment. This difference indicates that there are implications for how universal nutrition programmes are evaluated: aggregate estimates of programme effectiveness may obscure important heterogeneity across both SES groups and developmental domains, potentially understating the equity challenge.

### Implications for practice and research

These findings have implications for the design and evaluation of nutrition programmes, particularly in LMICs. The observation that cognitive associations were broadly distributed across SES groups among girls, while mental health associations were concentrated among higher-SES families, suggests that ‘one-size-fits-all’ biomedical interventions, although necessary, may be insufficient on their own for achieving equitable developmental outcomes. For policymakers in such settings, this reframes the core question from ‘whether to supplement’ to ‘how to implement supplementation more equitably’. To prevent such programmes from inadvertently widening health disparities, nutritional support should be integrated with targeted psychosocial measures for vulnerable families, including parenting education, social support services, and community-based mental health resources. An integrated approach is essential for translating the biological potential of early nutrition interventions into equitable long-term developmental gains, in alignment with SDG 3 (Good Health and Well-Being) and SDG 10 (Reduced Inequalities).

Beyond programme design, our findings add to the evidence base supporting folic acid supplementation as a cost-effective early childhood development strategy, with potential benefits extending beyond NTD prevention [44,45]. Public health messaging may therefore benefit from moving beyond traditional NTD-focused communication to highlight broader neurodevelopmental and mental health dimensions. Emphasising that maternal folic acid supplementation is associated with long-term benefits for health and human capital, even for children without birth defects, could provide WRA with additional motivation for adherence, particularly in resource-constrained settings where awareness of these longer-term benefits remains limited.

Methodologically, the complementary integration of macro-level health surveillance data (MCHS) and micro-level nationally representative longitudinal data (CFPS) within a quasi-experimental framework offers a replicable model for evaluating long-term policy impacts. Dynamic disease surveillance networks enable policymakers to monitor health burdens in real time, while investment in nationally representative longitudinal cohorts allows researchers to track long-term human capital accumulation and uncover hidden socioeconomic disparities. Investing in analogous dual-track data infrastructure could meaningfully strengthen evidence-based policymaking in LMICs.

Finally, although the Chinese experience offers a reference point for other LMICs considering large-scale folic acid supplementation, direct policy transfer requires caution. China’s institutional infrastructure, unique demographic history, and the specific social policy landscape of the study period, including concurrent gender-equity initiatives, may not be replicable elsewhere. Contextual adaptation, rather than wholesale extrapolation, is the appropriate posture when drawing on these findings in other national settings.

### Strengths and limitations

This study had several notable strengths. First, our analyses relied on professionally administered cognitive tests and validated, standardised mental health instruments. This rigorous approach yielded robust, objectively measured outcomes, mitigating the reporting and recall biases inherent in the parent-reported proxies frequently utilised in existing studies. Although the 2020 CFPS wave did not contain objective tests for language or behavioural outcomes, we conducted supplementary analyses using available parent-reported proxy measures (online Supplemental Table S5). Second, by evaluating a large-scale, nationwide public health programme through a quasi-experimental framework, our findings captured real-world policy associations at the population level, offering insights that are difficult to obtain from small-scale controlled trials. Third, a primary methodological strength of this study was the innovative linkage of nationally representative micro-survey data (CFPS) with restricted-access, macro-administrative data (MCHS), providing a replicable methodological blueprint for long-term policy evaluation.

Several limitations should be noted. Folic acid exposure was measured indirectly using provincial-level NTD prevalence as a proxy for intervention intensity, precluding individual-level inference. The mixed-mode survey design introduced potential bias: girls in the 10–15 age group tend to be stronger verbal communicators than boys [46,47], which may have systematically favoured female participants in telephone-based mental health assessments, and the low face-to-face interview completion rate (16%) constrained the cognitive analysis sample. Additionally, unmeasured confounders, including maternal health-seeking behaviour, dietary quality, child nutritional intake, and household food security, may account for part of the observed associations. The absence of data on specific nutrients, such as iron, iodine, and protein, precluded disentangling the contribution of folic acid from the broader nutrition environment. Finally, we were unable to disentangle the precise biological and psychosocial mediating pathways [48–52]; therefore, future mechanistic studies with comprehensive clinical data would be warranted.

## Conclusions

Leveraging China’s 2009 Folic Acid Supplementation Programme for the Prevention of Neural Tube Defects as a quasi-experimental framework, this study identified sex-specific associations between programme-level folic acid exposure intensity and girls’ long-term cognitive and mental health outcomes at ages 10–15. The findings should be interpreted within the broader nutritional ecology and wider macro-social context of the study period, rather than the isolated biological effects of a single micronutrient.

Within this interpretive frame, we identified a critical equity dimension. While cognitive associations were broadly distributed among girls regardless of socioeconomic background, mental health associations were concentrated exclusively among those from higher-SES families. This suggests that standalone biomedical interventions, even when universally and freely provided, risk inadvertently concentrating certain benefits among more advantaged groups and widening mental health inequalities. Future public health strategies should move beyond a purely biomedical approach and integrate nutrition programmes with targeted psychosocial support for socioeconomically vulnerable families. This would help ensure that the potential dividends of public health investments are more equitably shared and contribute to breaking the intergenerational cycle of disadvantage.

## Supplementary Material

Supplementary file.docx

## Data Availability

The micro-level data from the China Family Panel Studies used in this study are publicly available upon registration and data access application at the Peking University Open Research Data Platform (https://cfpsdata.pku.edu.cn/#/home). The macro-level neural tube defect (NTD) prevalence data sourced from the Maternal and Child Health Surveillance system (http://www.mchscn.cn/) are restricted-access administrative data and are not publicly available due to data protection regulations. However, the aggregated provincial-level NTD prevalence data used as the policy proxy in our analyses are fully provided in the online Supplementary Material (online Supplemental Table S1).
