# Supplementary material for: The equity and effectiveness of China’s 2009 folic acid supplementation programme for long-term child development
Source: Glob Health Action. 2026 Jun 22;19(1):2691381. doi: 10.1080/16549716.2026.2691381 (PMC13288903; doi:10.1080/16549716.2026.2691381)
Supplement: Supplementary file.docx [file ZGHA_A_2691381_SM2817.docx]

# The equity and effectiveness of China’s 2009 Folic Acid Supplementation Programme for long-term child development

# Appendix A. Supplementary material

**Table S1.** Prevalence of neural tube defects (NTDs) by province in China, 2009 (per 10,000 births).

|  | *n* | Mean | SD | p25 | p50 | p75 |
| --- | --- | --- | --- | --- | --- | --- |
| ${NTD}_{09}$ | 24 | 5.67 | 3.62 | 2.25 | 5.12 | 8.48 |

SD, standard deviation.

p25, 25th percentile.

p50, 50th percentile.

p75, 75th Percentile.

**Table S2.** Pre-trend test of cognitive ability and mental health outcomes in Chinese children aged 10–15 years.

|  | Coefficient | Model^a^ (95% CI) | | *p*-value |
| --- | --- | --- | --- | --- |
| **Memory test score** | 0.026 | −0.017 | 0.070 | 0.235 |
| **Number series test score** | 0.276 | −0.431 | 0.984 | 0.442 |
| **CES-D20 score** | 0.026 | −0.041 | 0.094 | 0.439 |
| **Depressive symptoms** | 0.003 | 0.000 | 0.007 | 0.086 |

CI, confidence interval.

^a^ Adjusted for maternal age, paternal age, maternal education, and paternal education.

**Figure S1.** Trendline figures of outcome variables in policy-eligible and policy-ineligible areas.


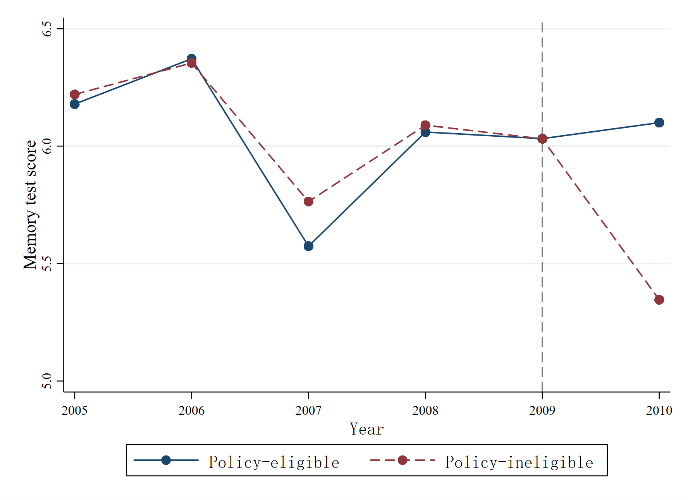

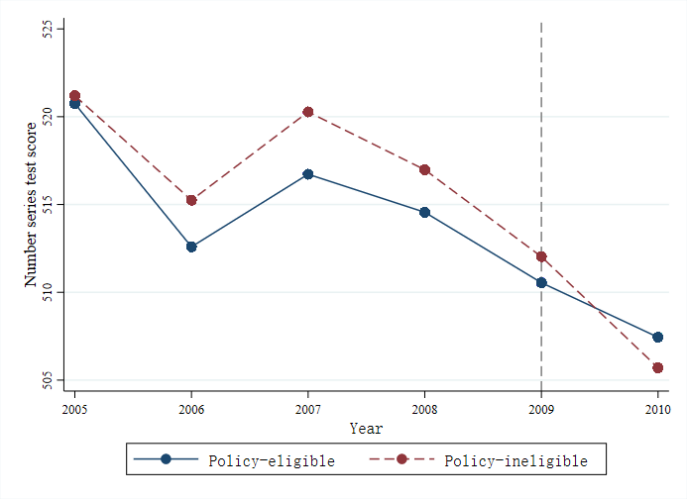

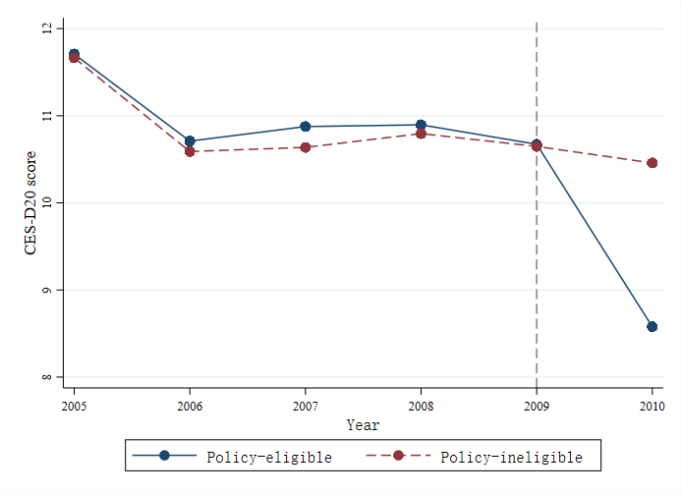

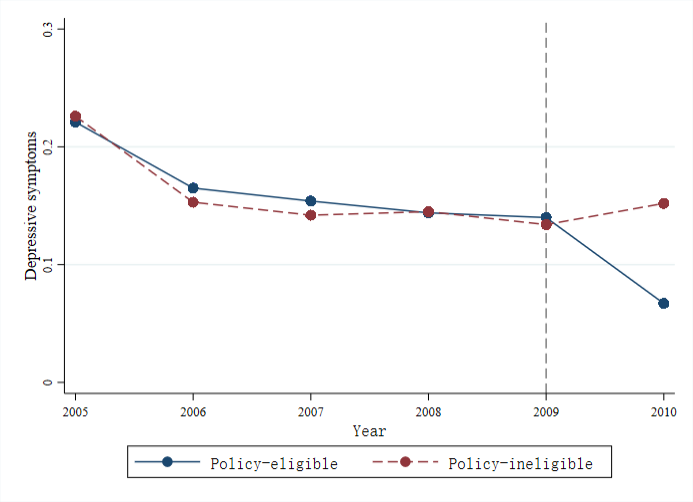


**Table S3.** Association of maternal folic acid supplementation with cognitive ability and mental health outcomes in Chinese girls aged 10–15 years, sensitivity analyses.

|  | Memory test score | | |  | Number series test score | | |
| --- | --- | --- | --- | --- | --- | --- | --- |
|  | *n* | Model 2^a^ (95% CI) | *p*-value |  | *n* | Model 2^a^ (95% CI) | *p*-value |
| Pre-intervention cohort (2006–09) | 145 | 0.242 | 0.006 |  | 146 | 5.186 | 0.001 |
|  |  | (0.071, 0.414) |  |  |  | (2.340, 8.032) |  |
|  |  |  |  |  |  |  |  |
| Intervention in 2006 | 102 | 0.047 | 0.662 |  | 103 | 3.253 | 0.155 |
|  |  | (−0.169, 0.263) |  |  |  | (−1.283, 7.790) |  |
|  | CES-D20 score | | |  | Depressive symptoms | | |
|  | *n* | Model 2^a^ (95% CI) | *p*-value |  | *n* | Model 2^a^ (95% CI) | *p*-value |
| Pre-intervention cohort (2006–09) | 781 | −0.550 | 0.011 |  | 781 | −0.028 | 0.014 |
|  |  | (−0.971, −0.129) |  |  |  | (−0.051, −0.006) |  |
|  |  |  |  |  |  |  |  |
| Intervention in 2006 | 602 | 0.135 | 0.323 |  | 602 | 0.010 | 0.201 |
|  |  | (−0.134, 0.404) |  |  |  | (−0.006, 0.026) |  |

CI, confidence interval.

^a^ Adjusted for maternal age, paternal age, maternal education, and paternal education.

**Table S4.** Risk ratios of depressive symptoms associated with maternal folic acid supplementation in Chinese children aged 10–15 years.

|  |  | Depressive symptoms | | | | |
| --- | --- | --- | --- | --- | --- | --- |
|  |  | *n* | Model 1 Risk ratio (95% CI) | *p*-value | Model 2^a^ Risk ratio (95% CI) | *p*-value |
| All |  | 1,953 | 0.888 (0.826, 0.955) | 0.001 | 0.891 (0.827, 0.960) | 0.003 |
| Boys |  | 1,005 | 0.927 (0.819, 1.049) | 0.231 | 0.938 (0.825, 1.067) | 0.331 |
| Girls |  | 918 | 0.843 (0.727, 0.978) | 0.024 | 0.851 (0.738, 0.981) | 0.026 |

CI, confidence interval.

^a^ Adjusted for maternal age, paternal age, maternal education, and paternal education.

**Table S5.** Association of maternal folic acid supplementation with parent-reported behavioural and educational outcomes in Chinese children aged 10–15 years.

|  | Parent-child conflict frequency^b^ (per month) | | |  | Parent-reported Chinese performance^b^ (1–4) | | |  | Parent-reported Maths performance^b^ (1–4) | | |
| --- | --- | --- | --- | --- | --- | --- | --- | --- | --- | --- | --- |
|  | *n* | Model 2^a^ (95% CI) | *p*-value |  | *n* | Model 2^a^ (95% CI) | *p*-value |  | *n* | Model 2^a^ (95% CI) | *p*-value |
| All | 1,938 | −0.069 | 0.278 |  | 1,922 | 0.002 | 0.900 |  | 1,874 | 0.020 | 0.182 |
|  |  | (−0.195, 0.056) |  |  |  | (−0.036, 0.041) |  |  |  | (−0.010, 0.050) |  |
|  |  |  |  |  |  |  |  |  |  |  |  |
| Boys | 1,020 | −0.020 | 0.780 |  | 1,012 | −0.006 | 0.819 |  | 991 | 0.004 | 0.833 |
|  |  | (−0.162, 0.122) |  |  |  | (−0.058, 0.046) |  |  |  | (−0.035, 0.044) |  |
|  |  |  |  |  |  |  |  |  |  |  |  |
| Girls | 918 | −0.058 | 0.484 |  | 910 | 0.016 | 0.502 |  | 883 | 0.043 | 0.117 |
|  |  | (−0.222, 0.106) |  |  |  | (−0.032, 0.064) |  |  |  | (−0.011, 0.098) |  |

CI, confidence interval.

^a^ Adjusted for maternal age, paternal age, maternal education, and paternal education.

^b^ Estimates are from the same DID specification as in the main analysis. Higher scores on the Chinese and Maths performance scales indicate better parent-rated academic performance (1 = poor, 2 = fair, 3 = good, 4 = excellent). These outcomes are based on parent-reported proxy measures and should be interpreted with caution given their susceptibility to reporting and recall biases.

## Sensitivity analyses

To examine the robustness of our findings, we conducted several sensitivity analyses. These analyses assessed the consistency of the observed associations between folic acid supplementation and the outcomes under varying assumptions.

## Sensitivity to a different pre-intervention cohort choice

Tables 2 and 3 in the main text present results from a difference-in-differences (DID) design, in which the post-intervention cohort refers to children born in 2010, and the pre-intervention cohort includes children born between 2005 and 2009. Given the age difference between 10- and 15-year-old children, the selection of the pre-intervention cohort may have influenced the results. To evaluate the sensitivity of our findings, we examined an alternative pre-intervention cohort consisting of children born between 2006 and 2009. The associations were similar in direction and magnitude across the different pre-intervention cohort choices (Supplementary Table S3).

When redefining the pre-intervention cohort as children born during 2006–2009, we observed consistent positive associations for cognitive ability outcomes (memory test score: 0.242 [0.071, 0.414], *p* = 0.006 and number series test score: 5.186 [2.340, 8.032], *p* = 0.001), and inverse associations for mental health outcomes (CES-D20 score: −0.550 [−0.971, −0.129], *p* = 0.011; depressive symptoms: −0.028 [−0.051, −0.006], *p* = 0.014), aligning with the primary analyses. The results are robust to different choices of pre-intervention cohorts, indicating that our findings were not sensitive to the specific selection of the pre-intervention cohort period.

## Placebo test

A placebo test was conducted to assess whether the associations were attributable to the intervention rather than preexisting trends. For this test, we advanced the intervention start date to June 2006, three years earlier than the actual policy commencement in 2009, and replicated the analyses presented in Tables 2 and 3 (Supplementary Table S3).

No clear associations were observed for the cognitive ability outcomes (memory test score: 0.047 [−0.169, 0.263], *p* = 0.662; number series test score: 3.253 [−1.283, 7.790], *p* = 0.155) or mental health outcomes (CES-D20 score: 0.135 [−0.134, 0.404], *p* = 0.323; depressive symptoms: 0.010 [−0.006, 0.026], *p* = 0.201). These findings suggest that the results in Tables 2 to 5 correspond to the actual policy implementation period.

## Poisson pseudo-maximum likelihood regression results

The Poisson pseudo-maximum likelihood regression analyses indicated inverse associations between maternal folic acid supplementation and depressive symptoms in Chinese children aged 10–15 years (Supplementary Table S4). In the adjusted model (Model 2), among rural children born after 2009, each unit increase in folic acid intervention intensity (${NTD}_{09}$) corresponded to a risk ratio of 0.891 (95% CI: 0.827, 0.960; *p* = 0.003). The association varied by sex: for girls, the risk ratio was 0.851 (95% CI: 0.738, 0.981; *p* = 0.026), while for boys, it was 0.938 (95% CI: 0.825, 1.067; *p* = 0.331).
